# With Special Reference to the Children of To-Day

**Published:** 1921-01-22

**Authors:** 


### THE AFTER-EFFECTS OF INFLUENZA.

### With Special Reference to the Children of To-day.

The subject of influenza, or, more correctly, the outbreaks which have passed under that name, have received an amount of popular attention bearing almost an inverse ratio to the number of undisputed scientific facts known about the disease. But, as time passes, it has been gradually possible to collect from statistical and institutional sources a number of interesting details concerning the direct effects of influenza upon child-life in both this country and America, and, startling though some of the earlier crude mortality figures were, it is now of more practical interest to the health worker to realise what were the effects on those—and particularly on children—who did not die. In future it is to be hoped that outbreaks such as those of 1917-1919 will not recur, but we must expect repetitions in a milder form, and therefore be prepared with a thorough appreciation of their possible effect. The figures quoted later have been derived from a variety of sources, and although, in the personal experience of individual readers, one or other of our estimates may appear inaccurate, we would point out that in so short a sketch it is possible to generalise only, and this we have done with every effort to obtain a fair and average view of the situation.

### The Age Incidence, &c.

As a general classification we may take childhood to include all ages up to about thirteen years, and a survey of the age incidence of influenza between these limits shows that in the earlier epidemics a maximum occurred at three to four years, whereas subsequently this point moved gradually towards those about one year younger. It is noteworthy that, among children, more than half the cases appear to have occurred at ages of less than five years, and, whichever locality or outbreak we take, there is a decided similarity between age-incidence statistics. In any case, a fair summary may be made by saying that there was a marked selection towards children up to five years, whereas the very young infant under one or two months of age was relatively immune.

As might be expected, curves representing the pneumonias developed and the mortality figures follow very closely those of simple incidence, and it is unnecessary to give them in detail. But it is important to realise that the percentage deaths among these children reached an extremely high figure when compared with the average for all ages. Above our stated age-limit mortality varied between five and 20 per cent. but below it, and particularly round about four years, a death-rate of even 40 to 50 per cent. was sometimes reached. But the exact proportion of children infected at any time is difficult to ascertain, owing to deficient or erroneous notification, the difficulties attending accurate diagnosis at these times, and the considerable number of cases nursed at home. Therefore we have confined ourselves to institutional figures, and those who, during the outbreaks, were in touch with children's hospitals or wards will realise what the high mortality meant in loss of child-life. We are aware that in certain epidemics, notably some in London, the maximum loss appeared among adolescents, but again let us state that we are considering English-speaking countries in general.

### Complications.

But apart from deaths, there is the question as to how much damage influenza has done to those who recovered. To start with a frequently affected and important system of the body let us consider aural complications. Enlargement of the cervical glands, general pharyngeal adenitis and varying degrees of acute tonsillitis occurred in, roughly, 25 per cent. of the cases. In many instances these attacks laid the foundation of chronic glandular trouble and its sequelæ. Actual otitis media occurred in more than two per cent., and without doubt many cases of defective hearing will be ultimately blamed back to influenza. Middle ear infections and acute mastoiditis became, too, more frequent in the later epidemics, so that the actual damage may, by figures, be more than we have quoted.

Turning next to the most deadly complication—namely, pneumonia—it is unnecessary to detail all the possible after-effects of an extreme degree of lung involvement. By far the greater number of child cases showed the broncho-pneumonic type of distribution and the condition, at the worst period, developed in over 65 per cent. of them. Apart from the fact that at times nearly one-half of these pneumonic children died, the possible events during the convalescence and up-growing of the rest will be readily appreciated. Pleurisy and empyema (two per cent.), laryngitis with intubation and tracheotomy in a few instances, all occurred with varying frequency and numerous cases of delayed resolution in the inflamed lungs went to swell the list of those children whose development has been either retarded or impaired.

### The Liability to Tubercle.

One curious point is that, as far as can be ascertained, these influenzal outbreaks have not, generally speaking, been a direct cause of any great increase in the subsequent manifestation of tuberculosis infections. It may be that those most likely to be infected with tubercle or any other disease were those weaker ones who died. On the other hand, physicians may have been more on the look out for true post-influenzal or post-catarrhal glandular conditions, and their differentiation from tuberculosis have been more complete. In any case it is a noticeable fact that many, if not all, statistics from the various countries and hospitals affected show if anything a decrease in the number of tuberculous children encountered within twelve months of the last epidemic.

"GASTRIC INFLUENZA."

The persistent vomiting which occurred in some juvenile cases is well enough recognised. Jaundice of the catarrhal type occasionally appeared, and frequently severe colitis and enteritis formed a recognised and dangerous complication. But more interesting from our particular standpoint of the moment is the fact that a number of these cases have showed varying degrees of post-epidemic vomiting and gastro-intestinal irritability of a degree sufficiently marked to require careful watching if normal development was to be maintained. Generally practitioners will agree that they have been readily amenable to careful nursing, but the typical post-influenzal child showing an acidosis and persistent leucocytosis in the blood is by no means rare even to-day.

RARER INSTANCES AND EXCEPTIONS.

Nephritis has been so infrequently seen that it is impossible to give a figure for it, but meningitis, generally with fatal endings, can be recorded in about one per cent. of the cases. Of these latter and the occasional encephalitic conditions we cannot here discuss the possible and severe effects upon a survivor's after-life. More interesting is the relative scarcity of true endocarditis or pericarditis, in spite of the undoubtedly virulent bacterial infections which were present. Even the more extreme degrees of myocarditis were not often severe in children, and, although we know ourselves of one or two exceptions, generally speaking the influenzal outbreaks did directly relatively little permanent damage to the young sufferers' hearts. Sir James Mackenzie has never seen any case of influenza in which damage was limited to the heart alone, as in rheumatic fever. Although the cardiac involvement occurred along with the lung complications, he states that he has never known a patient recover with damaged valves.

MENTAL EFFECTS.

According to Sir George Savage, influenza, of all infectious diseases, is the most likely to be followed by mental disorder, which as a rule is of a favourable type for recovery. Unfortunately, from his experience, early youth has been one of the most dangerous and most likely periods in which this complication may occur, and we have already mentioned the occurrence of direct meningeal infection. There is little doubt that backwardness will often arise directly and indirectly from these causes, and it must be classed among the rarer sequelæ to influenza. But enough has been outlined to illustrate the necessity that we should in future, when taking a case history, remember the possible happenings to a child during the influenza years, and also the fact that these catarrhal outbreaks have national effects even more far-reaching than the number of deaths which they immediately caused.

